# Xenon-Enhanced Dynamic Dual-Energy CT Is Able to Quantify Sinus Ventilation Using Laminar and Pulsating Air-/Gas Flow Before and After Surgery: A Pilot Study in a Cadaver Model

**DOI:** 10.3389/falgy.2022.829898

**Published:** 2022-02-16

**Authors:** Sven Becker, Tilman Huppertz, Winfried Möller, Miriam Havel, Maria Schuster, Anne Merle Becker, Martin Sailer, Uwe Schuschnig, Thorsten R. Johnson

**Affiliations:** ^1^Department of Otolaryngology, Head and Neck Surgery, Tübingen University Hospital, Tübingen, Germany; ^2^Department of Otorhinolaryngology, Head and Neck Surgery, University Medical Centre, Johannes Gutenberg University Mainz, Mainz, Germany; ^3^Institute of Lung Biology and Disease, Helmholtz Center München, Helmholtz Association of German Research Centres (HZ), Munich, Germany; ^4^Department of Otorhinolaryngology, Head and Neck Surgery, Ludwig-Maximilians-University, Munich, Germany; ^5^Pari Pharma GmbH, Gräfelfing, Germany; ^6^Institute for Clinical Radiology, Ludwig-Maximilians-University, Munich, Germany

**Keywords:** chronic rhinosinusitis, CRS, dual energy CT, xenon, ventilation imaging, sinus ventilation, pulsating airflow

## Abstract

**Background:**

Chronic rhinosinusitis is a common disease with a significant impact on the quality of life. Topical drug delivery to the paranasal sinuses is not efficient to prevent sinus surgery or expensive biologic treatment in a lot of cases as the affected mucosa is not reached. More efficient approaches for topical drug delivery are, therefore, necessary. In the current study, dual-energy CT (DECT) imaging was used to examine sinus ventilation before and after sinus surgery using a pulsating xenon gas ventilator in a cadaver head.

**Methods:**

Xenon gas was administered to the nasal cavity of a cadaver head with a laminar flow of 7 L/min and with pulsating xenon-flow (45 Hz frequency, 25 mbar amplitude). Nasal cavity and paranasal sinuses were imaged by DECT. This procedure was repeated after functional endoscopic sinus surgery (FESS). Based on the enhancement levels in the different sinuses, regional xenon concentrations were calculated.

**Results:**

Xenon-related enhancement could not be detected in most of the sinuses during laminar gas flow. By superimposing laminar flow with pulsation, DECT imaging revealed a xenon wash-in and wash-out in the sinuses. After FESS, xenon enhancement was immediately seen in all sinuses and reached higher concentrations than before surgery.

**Conclusion:**

Xenon-enhanced DECT can be used to visualize and quantify sinus ventilation. Pulsating air-/gas flow was superior to laminar flow for the administration of xenon to the paranasal sinuses. FESS leads to successful ventilation of all paranasal sinuses.

## Introduction

Chronic rhinosinusitis (CRS) is a common disease affecting approximately 15% of the population in western countries (1, 2). Conservative treatment options include topic or systemic corticosteroids, oral long-term antibiotics, and nasal douche. In more recent years, biological treatment for severely affected patients is a highly effective new opportunity (3). However, the number of surgical interventions in the patients with CRS is high. Apart from medical history and nasal endoscopy, CT of the paranasal sinuses is considered as the gold standard to diagnose CRS (4). CT images provide a precise impression of the bony structures and the surrounding tissues, for example swelling of the lining mucosa (5). On the other hand, no functional analysis for instance of sinus ventilation is possible, when standard CT protocols are used. To better estimate treatment success, knowledge about sinus ventilation could be helpful when using topical corticosteroids in form of an aerosol generated by a nebulizer. Paranasal sinuses are non-actively ventilated cavities were local deposition of drugs remains challenging. To improve gas and aerosol transport into the sinuses pressure gradients between the two sides of the ostia are necessary (6, 7). This effect can be attained with a pulsating airflow generated by nebulization devices (7–11).

The introduction of dual-source CT systems [dual-energy CT (DECT)] has improved material differentiation. This is achieved by different tube voltages which are able to generate different X-ray energy spectra (4, 12). Radiopaque stable xenon gas leads to an increased absorption with decreasing photon energies as a result of photoelectric interactions (atomic number of stable xenon gas Z054) (13). Furthermore, the concentration of xenon is linearly associated with its X-ray attenuation (14). Hereby, selective xenon gas visualization can be reached. Furthermore, xenon wash-in and wash-out dynamics using successive CT datasets can be provided. Earlier studies investigated the efficiency of xenon as a CT contrast agent by multiple CT dataset acquisition measurements for the evaluation of xenon wash-in and wash-out characteristics in the paranasal sinuses (15, 16). Our working group could demonstrate sinus ventilation using pulsating gas flow by DECT and dynamic CT imaging in a rudimentary nasal plastic cast. Thus, the aim of the current pilot study was to visualize and quantify sinus ventilation using laminar and pulsating airflows in a cadaver head by DECT before and after surgery.

## Methods

### Cadaver Head

For the ventilation examinations, a formalin-fixed female cadaver head from the Anatomical Institute of the Ludwig-Maximilians University, Munich, Germany was used. The mandible with tongue and floor of the mouth was removed to get a better access to the nasopharynx which was occluded by a silicon plug. Endoscopic evaluation revealed no significant septal deviation or any signs of previous surgical procedures on the paranasal sinuses. The study was approved by the local ethics committee.

After the first measurement without manipulation, endoscopic sinus surgery was performed on the cadaver head. Following standard approach of functional endoscopic sinus surgery (FESS), an operation on all sinuses was performed. Thereafter, a second measurement was performed.

### Xenon Application System

**Figure 1:**
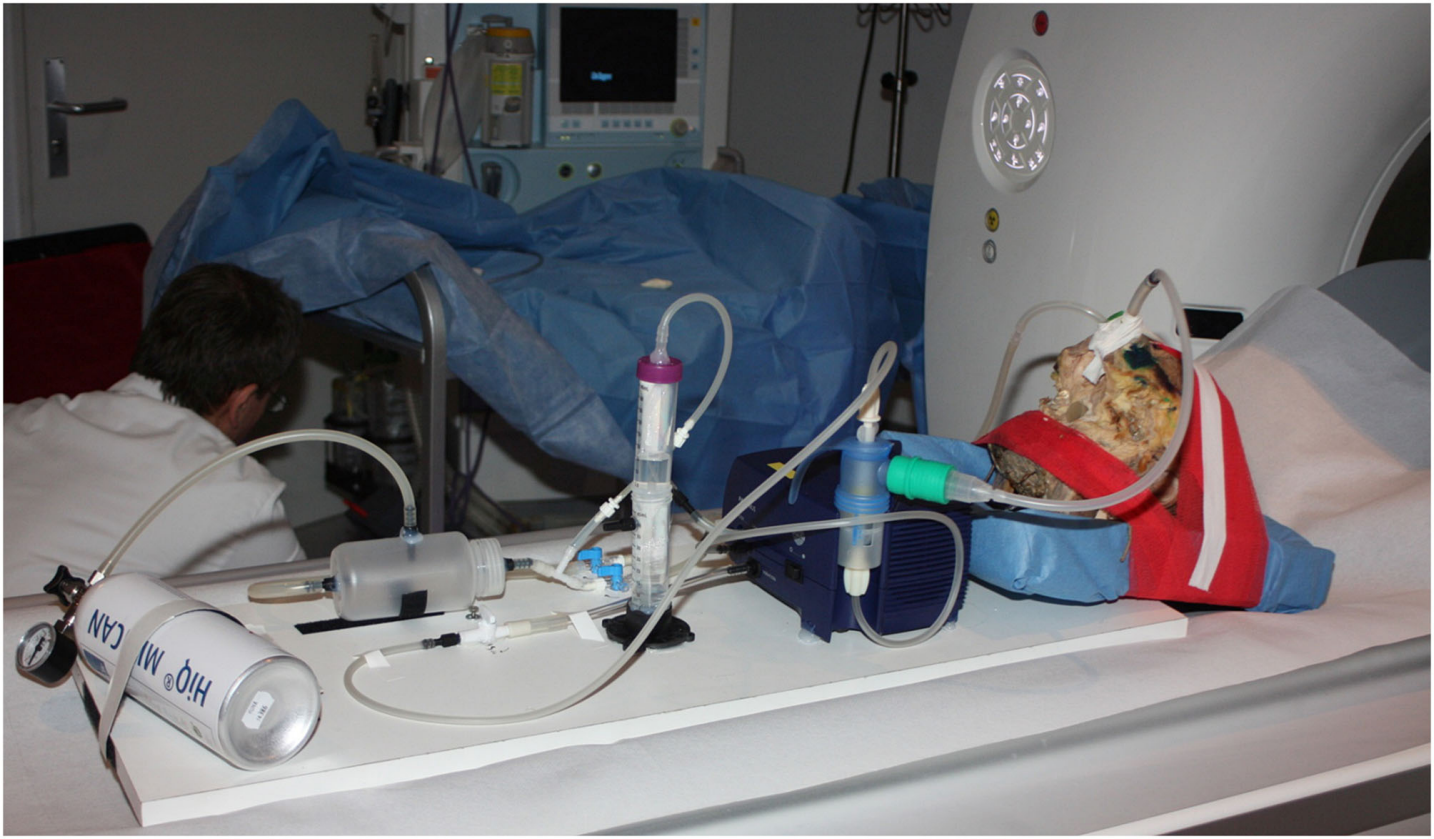
Experimental setting. Fixated cadaver head with connected xenon supplying nebulization system (blue nebulizer + xenon tank and gas recirculation system) on the patient table of the SOMATOM CT system.

A pulsating airflow was generated using the PARI SINUS system (Pari GmbH, Starnberg, Germany), which is based on a PARI BOY (PRONEB Ultra in the USA) aerosol drug delivery device. The system includes a compressor with an integrated pressure wave generator. It produces amplitude of 25 mbar with a frequency of 45 Hz. The device was connected to a tank with 100 % xenon (Linde, Munich, Germany, purity 99.996 %). The gas flow rate was 7 L/min in both settings with and without pulsation. The whole setup was coupled to both nostrils of the cadaver head. Xenon gas and pulsation were administered to the left nostril, returning gas from the right nostril was captured in a collecting tank and again insufflated *via* the left nostril (Figure 1). This way gas consumption could be decreased.

### Dual-Source CT System

A dual-source SOMATOM force CT system (Siemens Healthcare, Forchheim, Germany) was used. Cadaver head and xenon supplying nebulization system were positioned on the patient table of the SOMATOM CT system (Figure 1).

### DECT Examination

For xenon DECT measurements, 46 subsequent CT series were scanned at a frame rate of 1.5 s. The imaging range covered nostrils and the nasal cavity as well as the frontal, maxillary, and sphenoid sinuses. The examination was started with a laminar flow of room air, and imaging began while continuous alternating table movement. The laminar flow was switched from room air to 100% xenon after 10.5 s of imaging for 42 s. After 24 s of xenon supply, pulsation of xenon flow was started for 18 s. At 52.5 s, xenon influx and pulsation were stopped, and laminar flow with room air was continued. At 63 s after beginning of the examination, pulsation with room air was switched on to washout the xenon gas from the sinuses. The acquisition of images stopped at 69 s.

After performing FESS on the cadaver head, 33 subsequent CT series were captured at the above-mentioned frame rate covering the same anatomical structures as before surgery. Once more, the examination started with a laminar flow of room air in the course of continuous alternating table movement. Approximately, 10.5 s after starting the examination laminar room air flow was switched to laminar 100% xenon flow. Further 24 s later, pulsation was initiated until the end of the measurement. These measurements were abbreviated at 50 s, since complete ventilation with and without pulsation could be seen. The wash out phenomenon was, therefore, not documented.

The CT setup and DECT image reconstruction were comparable to the setup of a previous study in a rudimentary plastic cast model of our working group with following parameters (4): Tube voltage of tube A with a tube current–time product of 100 effective mAs was 100 kV; voltage of tube B (generating a hardened 140 kVp spectrum using a tin filter) and a tube current–time product of 85 effective mAs was 140 kV; slice collimation, 128 mm × 0.6 mm; rotation time, 0.28 s; pitch, 0.55 (4).

### Dual Energy CT Image Reconstruction

For the reconstruction of the acquired images, a soft kernel (B30f) at a slice thickness of 1 mm with 0.7-mm increment was used. Post-processing of the reconstructed image datasets from the two different energy tubes was performed after transferring the datasets to a syngo Multi Modality Workplace (Siemens Healthcare, Germany). Xenon enhancement was color-coded by a specific DE post-processing software. Afterward, these enhancement maps were fused with the axial images (4).

### Data Analysis

**Figure 2:**
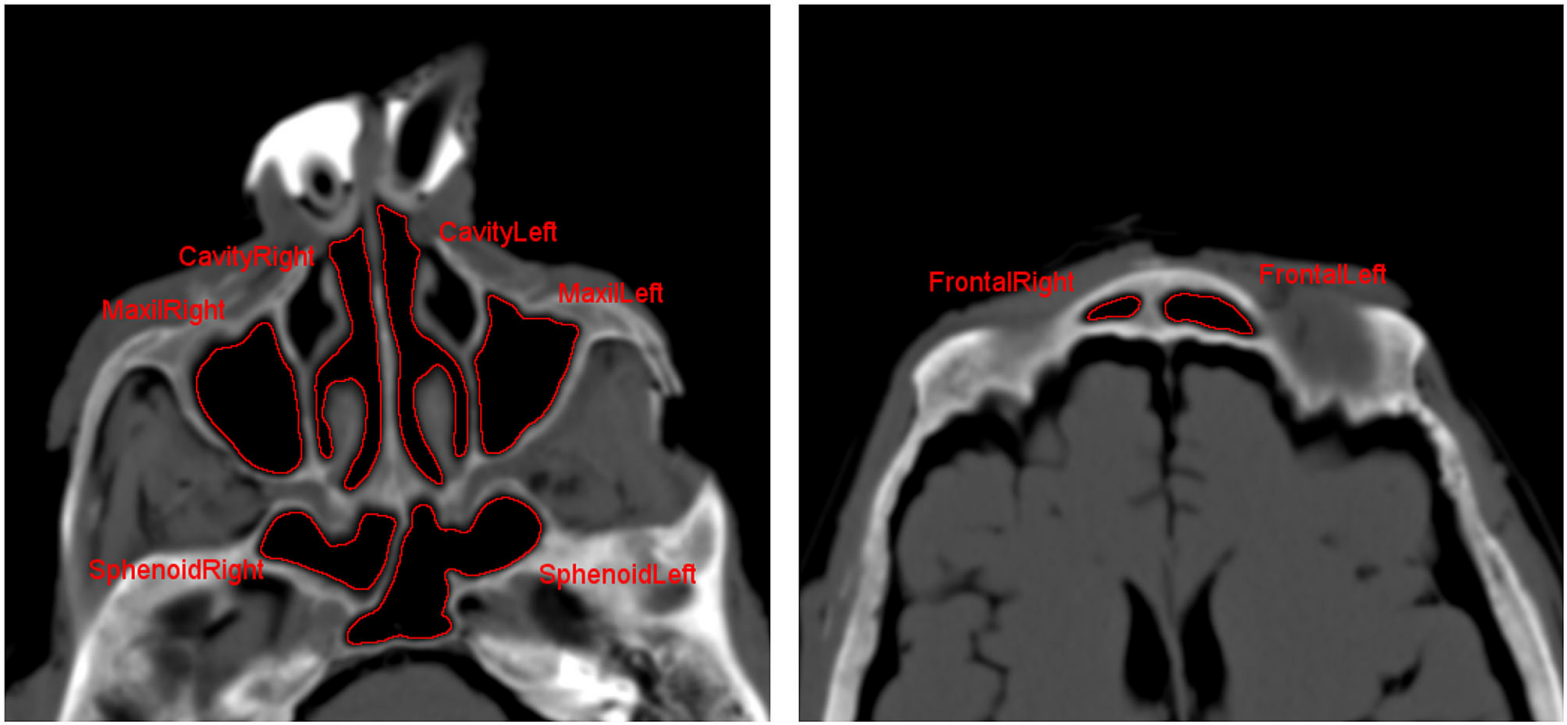
Axial CT scan for placement of regions of interest (ROIs) in nasal cavity as well as in the different paranasal sinuses.

Regions of interest (ROIs) were placed inside, both nasal cavities, and the different adjacent sinuses on both sides (frontal, sphenoid, and maxillary sinuses) (see Figure 2). Overall 46 xenon concentrations could be measured in each ROI with a time interval of 1.5 s. Hounsfield units (HU) were recorded from each imaging serie, and time–density curves were generated. Based on the time–density curves, a first-order exponential function was fitted, and the characteristic time constant for the xenon concentration change within the sinuses, τ, was determined for xenon wash-in and wash-out for each sinus separately (4, 16). The enhancement level was calculated by the difference between the HU values and −1,000 HU (value of room air). The enhancement value of 100% xenon was defined as the maximum enhancement within the input nostril. Xenon concentrations were determined for each time point and each ROI by calculating the ratio of the respective xenon enhancement and that of 100% xenon (4).

## Results

### Dual Energy CT Measurements in the Nasal Cavities

**Figure 3:**
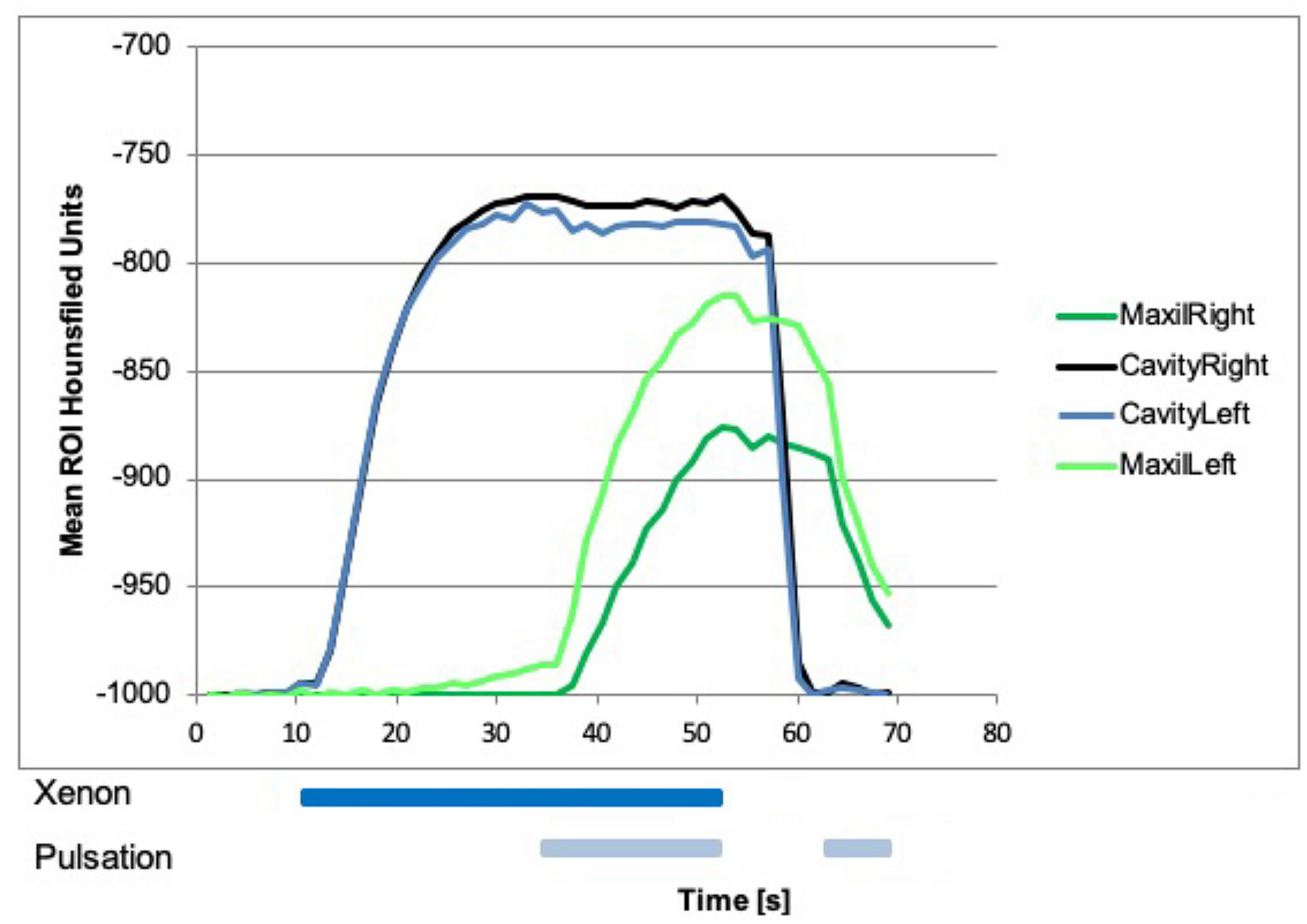
Xenon concentration in the nasal cavities, with a steep rise after influx of 100% xenon at 10.5 s on both sides (black and blue line). Start of pulsation had no effect on the concentration. Xenon influx was stopped at 52.5 s then a steep fall of xenon concentration in both nasal cavities could be seen. Xenon concentration in the maxillary sinuses with a rise after the influx of 100% xenon, and the start of pulsation at 34.5 s (green lines) could be seen. Influx of xenon without pulsation had no effect on the concentration in the maxillary sinuses. After that, xenon influx and pulsation were stopped at 52.5 s, the xenon concentration was slowly declined. With a renewed start of pulsation with room air at around 63 s, a steeper decline in both maxillary sinuses could be seen.

Beginning with laminar airflow with room air at 0 s and with 100% xenon from 10.5 s onward, a rise in xenon concentration could be detected in both nasal cavities reaching a plateau at around 20 s (Figure 3, blue and black line). At that time point, nasal cavity is filled with nearly 100% xenon gas corresponding to −750 HU. Room air has around −1,000 HU. The start of pulsation at 34.5 s had no further effect on the xenon concentration in the nasal cavity. After xenon influx was stopped and room air was again delivered at 52.5 s xenon was rapidly cleared out of both nasal cavities. A renewed start of pulsation at 63 s did not change the concentration (Figure 3, blue and black line).

### Dual-Energy CT Measurements in the Maxillary, Sphenoid, and Frontal Sinuses

**Figure 4:**
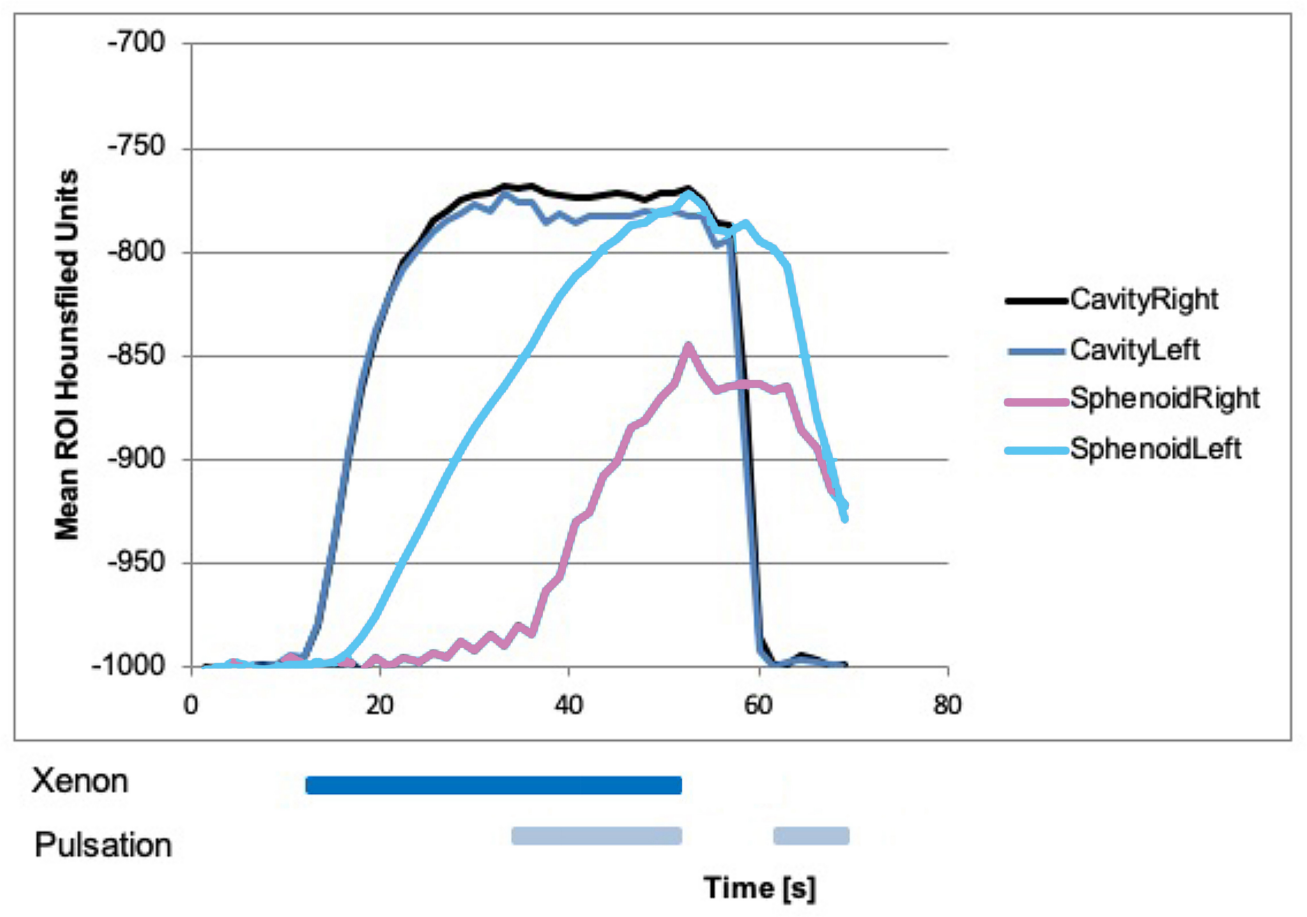
Xenon concentration in the sphenoid sinuses, with a rise in the left side after influx of 100% xenon and no difference on the right side. With the start of pulsation at 34.5 s, there was also a rise in the right side. After that, xenon influx and pulsation were stopped at around 52.5 s, the xenon concentration was slowly declined on both sides. With a renewed start of pulsation with room air at around 63 s, the steeper decline in both sphenoid sinuses could be seen.

**Figure 5:**
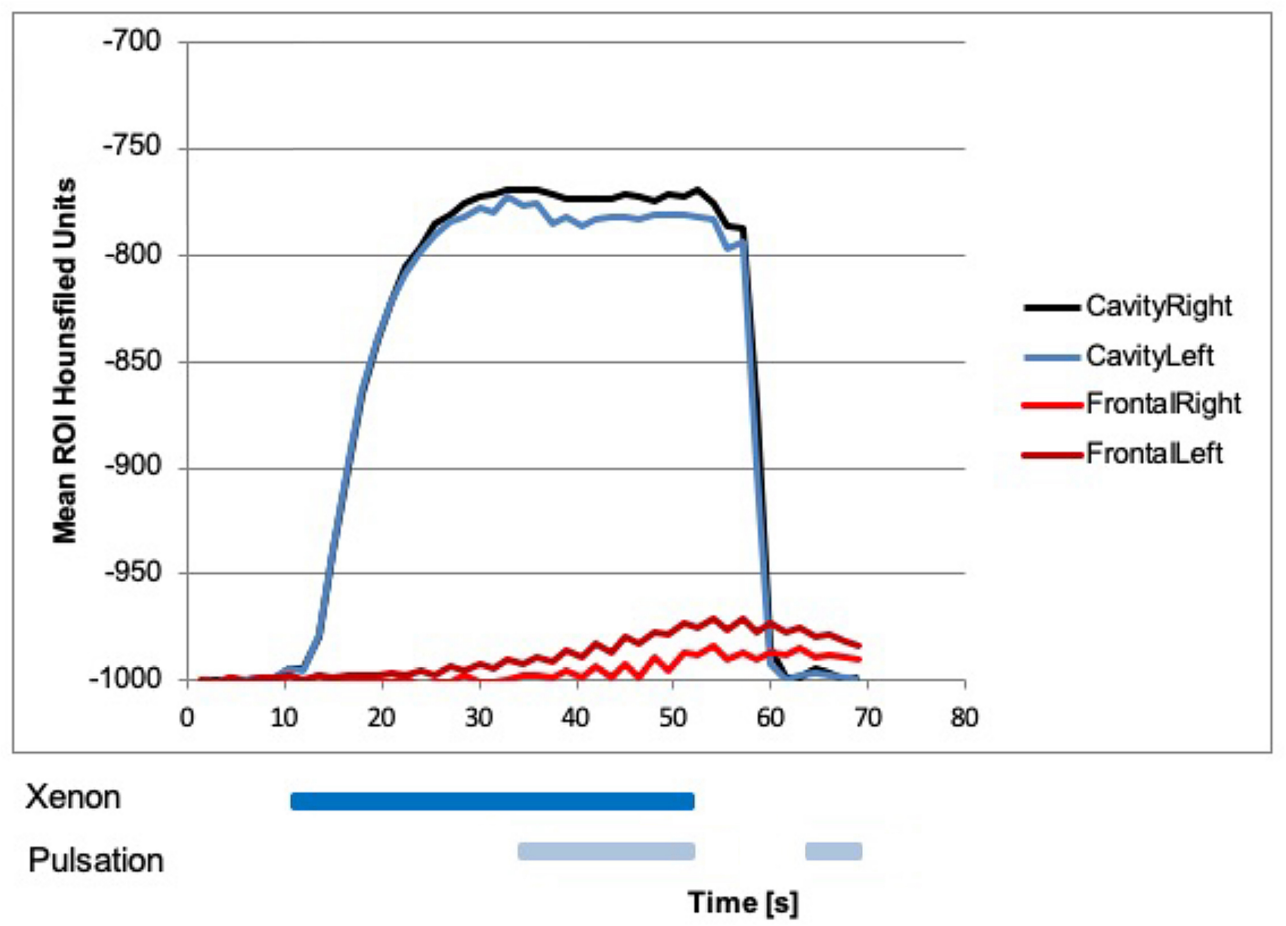
Xenon concentration in the frontal sinuses, with a rise after the influx of 100% xenon and start of pulsation at 34.5 s. Influx of xenon without pulsation had no measurable effect on the concentration in the frontal sinuses. After that, xenon influx and pulsation were stopped at around 52.5 s, the xenon concentration was slowly declined. With a renewed start of pulsation with room air at around 63 s, a further decline in both frontal sinuses could be seen.

Beginning with laminar airflow with room air at 0 s and with 100% xenon from 10.5 s onward, a rise in xenon concentration could be detected in the left sphenoid sinus beginning around 15 s (Figure 4). In the other sinuses, no rise in xenon concentration was registered (Figures 3–5). With the start of pulsation at 34.5 s, a steep rise could be seen in both maxillary sinuses and the right sphenoid sinus (Figures 3, 4). In the frontal sinuses, the rise was less pronounced but measurable (Figure 5). The concentration in the left sphenoid sinus showed a further rise.

After 52.5 s, xenon inflow and pulsation were stopped and laminar airflow with room air continued. The xenon concentration in all sinuses slowly declined. Approximately 63 s after the beginning of the experiment, pulsating airflow with room air was started again and a drop in xenon concentration in all paranasal sinuses could be seen, resembling an active washout. Again this was especially marked in the maxillary sinus with a τ of 6 s (Figure 3).

The absolute xenon concentrations reached are between 50 and 90% in the maxillary and sphenoid sinuses, with the sphenoid sinuses reaching slightly higher values. In the frontal sinuses, the maximum xenon concentration reached is around 10%.

Overall, the left-sided paranasal sinuses reached higher xenon concentrations and the rise in xenon concentration was faster, best seen in the maxillary sinus with a τ of 7 s on the left and a τ of 18 s on the right side (Figure 3).

### Dual-Energy CT Measurements in the Maxillary, Sphenoid, and Frontal Sinuses After FESS

**Figure 6:**
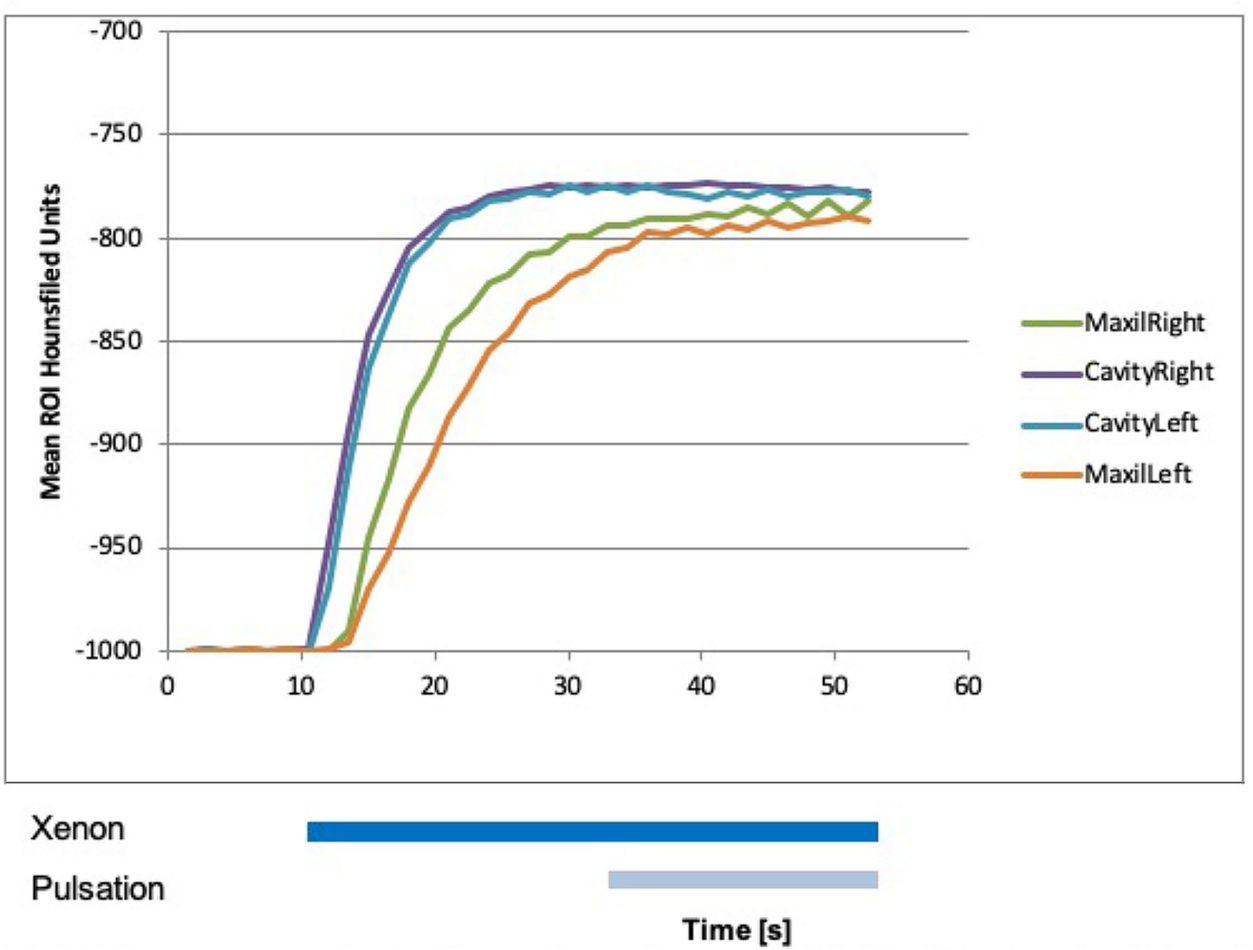
Xenon concentration in the maxillary sinuses after functional endoscopic sinus surgery (FESS), with a rise after the influx of 100% xenon at 10 s. The start of pulsation at 34.5 s did not change influx.

**Figure 7:**
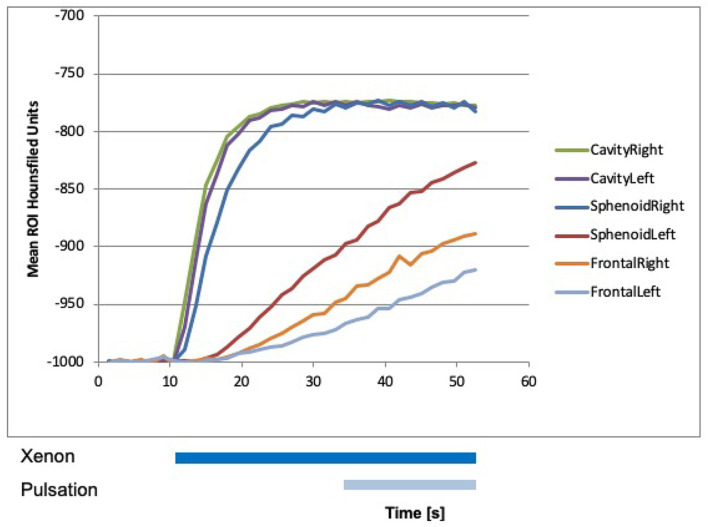
Xenon concentration in the frontal and sphenoid sinuses after FESS, with a rise after the influx of 100% xenon at 10 s. The start of pulsation at 34.5 s did not change influx.

After performing FESS, measurements were repeated. Beginning with laminar airflow with room air at 0 s and with 100% xenon from 10.5 s onward, a steep rise in xenon concentration could be detected in both nasal cavities, in the maxillary sinuses, and in the right sphenoid sinus reaching a plateau between 20 and 30 s (Figures 6, 7). The xenon concentration reached was around 90%. The start of pulsation at 34.5 s had no effect on the xenon concentration. In the left sphenoid sinus and the frontal sinuses, the increase in xenon concentration was delayed and not as marked. Still it was significantly higher than before surgery. This measurement was abbreviated at 50 s, so that washout was not documented.

## Discussion

In this study, we could show that visualization and quantification of sinus ventilation using laminar and pulsating air-/gasflow in a cadaver head is possible by xenon-enhanced DECT. Exchange phenomena of the contrast agent between the nasal cavity and the paranasal sinuses under laminar and pulsating xenon gas flow could be seen. Also, we could show that pulsating gas flow leads to xenon influx into the non-operated paranasal sinuses in contrast to laminar flow, similar to our results with a nasal cast and as described in the literature (4, 7, 9–11). By using pulsation, it was possible to transport xenon into the non-operated paranasal sinuses. Ventilation time constants of approximately 10 s showed a nearly two orders of magnitude faster distribution than passive diffusion. Hence, the effects of passive diffusion can be ignored when using a time schedule as applied in the current study.

Xenon-enhanced CT to evaluate the sinus ventilation was first described by Kalender et al. in 1985 (15). Sinuses were filled with Xenon by placing a ballon-tipped catheter in each nostril and positive pressure insufflation during intermittent apnea. Sinuses were then imaged by single energy CT during normal breathing and physiological washout rates of xenon were calculated. Further studies improving the existing protocol followed but were all focused on physiological ventilation while normal breathing of mainly the maxillary sinus (16–18). Paulsson et al. examined the influence of sinus surgery on xenon wash-out and could demonstrate that surgery leads to improved sinus ventilation with faster xenon wash-out from the sinuses (19). Also, Brumund et al. could show that surgical widening of the ostium of the maxillary sinus improves ventilation in a sheep model. Interestingly, a small antrostomy produced a statistically significant increase in maxillary sinus ventilation. No further significant increase was obtained by creating a large antrostomy (20). Beside us, no other working group used xenon-enhanced DECT to examine sinus ventilation during application of laminar and pulsating airflow before and after sinus surgery.

In the current study, the left sphenoid sinus showed uptake of xenon about 5 s after laminar flow with xenon began which is probably due to an anatomical variation with a sufficiently wide natural ostium and the fact that gas influx was given on the left side. This might have resulted in a direct flow of xenon into the left sphenoid sinus. Begin of pulsation did not influence further uptake of xenon into this sinus.

Only in the frontal sinuses xenon uptake under pulsation was very low. This might be explained by the anatomy of the frontal sinus with a canal of firm bone that might not be as easily accessed by gas and might also not meet criteria necessary for an exchange in adjacent compartments as described by Helmholtz due to the thickness of the surrounding bone making a vibration of this compartment difficult (21, 22). The so-called Helmholtz resonator is an acoustical device composed of a sphere cavity attached to an narrow tube also known as the neck (22). When this resonator is exposed to an external acoustic field, the air plug inside the neck oscillates at a frequency equal to that of the external field. The amplitude of the air plug in the neck oscillates according to the different frequencies of the external acoustic field. Maximum gas exchange between the cavity and the surrounding media occurs when the frequency of the external acoustic field equals the so-called “resonance frequency”, a specific frequency for each resonator (21–23). Transferred to the anatomy of the human head, each sinus with its ostium and the adjacent part of the nose (e.g., frontal sinus plus frontal recess) has its own resonance frequency due to form, thickness of bone, and mucosal properties. To reach all sinuses in an optimal way, a single frequency as applied in the current study is, therefore, not sufficient and can lead to enormous differences in sinus ventilation as seen between the maxillary and the frontal sinuses. To overcome this problem, future nebulization devices could use a frequency sweep from deep frequencies (45 Hz) as used in the current study to higher frequencies up to 300 Hz or more within a 2 min therapeutic inhalation to reach each individual resonance frequency of the different sinuses for a short time. Maniscalco et al. for example could show that the deposition of drugs on the wall of the maxillary sinus can be increased by 3-, 3.5-, and 4.4-fold when laminar nebulized aerosol flow to the nostril was superimposed by pulsation of 45, 120, and 200 Hz, respectively (24). Similarly, positive results could also be achieved by nebulizers using a pulsation of 100 Hz (25, 26). Pourmehran et al. tried to maximize drug deposition in a single-sided maxillary sinus model by optimally suit frequency, amplitude, and flow rate of applied 12 μm aerosol particles by controlled repeated measurements (27). They were able to increase drug delivery by 75-fold when using a frequency of 328 Hz with an amplitude of 126 dB re 20 μPa and a flow rate of 0.267 ml/min showing that further developments in nebulization devices could have the opportunity to substantially improve topical drug delivery. As most of the studies focus on the maxillary sinus, further studies covering the frontal sinus are necessary to get a better understanding of how this also surgically more difficult to address cavity can be sufficiently reached.

Drug delivery to the sinuses is not only influenced by pulsation parameters of the applied aerosol flow, but also influenced by the breathing patterns, size of the aerosol particles, how the nebulizer is connected to the nose (inclination of the nosepiece) and the anatomy of the nasal airway itself (26–28). Last-mentioned could be shown by Hosseini and Golshahi in anatomical 3D printed nasal airway models of 2-, 5- and 50-year old human subjects (29). In their study, a pulsating airflow was applied with 44.5 Hz frequency and 24 mbar amplitude comparable to our study. Drug delivery to the maxillary sinus in the adult subject increased 4-fold when using pulsation in comparison to a laminar airflow without pulsation. They could show that drug deposition in the anterior part of the nose in the two younger subjects was higher than that in the adult model. This leads to a decrease of 3–11% in drug deposition to the maxillary sinus, and a 25% decrease in lung deposition showing the effect of anatomy/age on sinus drug delivery. They could also show that a bidirectional breathing administration technique can significantly increase the paranasal drug delivery when pulsating airflow is used (29).

Although there are numerous experimental studies on different nebulization devices with pulsation properties, clinical studies on the effectiveness of this kind of drug application in CRS patients are missing. To our knowledge, there are only two registered studies comparing corticosteroid application *via* nasal spray with nebulization plus pulsation in CRS patients with (EudraCT-Nr. 2013-002414-12) and without nasal polyps (EudraCT-Nr. 2013-002421-30). The first results from the latter study were promising and providing estimates for the sample size calculations to conduct a pivotal study in the future (30).

Having the above-mentioned anatomical and functional parameters in mind diagnostic approaches covering these aspects are necessary to evaluate if a patient is suitable for topical sinus drug delivery by nebulization devices or not and to further improve the nebulization parameters itself. In the future, xenon-enhanced DECT could be used for this purpose in patients with CRS to functionally evaluate sinus ventilation properties and sinus anatomy at the same time. Furthermore, it could help to optimize the nebulization parameters in standardized models and pave the way for improved topical corticosteroid delivery. Earlier studies in healthy participants could demonstrate the feasibility of dynamic assessment of paranasal sinus ventilation using xenon-enhanced CT (15, 16, 18). But xenon has anesthetic properties in higher concentrations and is used as an inhalation general anesthetic agent for this reason (31, 32). Therefore, a systematic use of xenon at high concentrations for imaging the paranasal sinuses has to be carefully evaluated. Otherwise, deep inhalation of xenon is not necessary for imaging sinus ventilation, as patients should close their soft palate during nebulizer use. A former study examined maxillary sinus ventilation in a dynamic protocol over 30 min. Subjects had to breath 30% xenon and experienced side effects like nausea with vomiting or lightheadedness (16). As already proposed, a study design with a short ventilation time but higher xenon gas concentrations while closing the soft palate could reduce the above-mentioned side effects (4).

After FESS, there was a very efficient influx of xenon into all sinuses with the frontal sinuses showing the smallest uptake. By widening the ostia of the sinuses to a maximum extend by FESS, the sinuses became part of the directly ventilated areas like the nasal cavity. Pressure gradients between the two sides of the ostia to ventilate the sinus *via* resonance properties were no longer necessary, so pulsation did not change uptake anymore. Therefore, measurements were abbreviated after 50 s and wash out of xenon was not documented. These findings are in line with existing literature (33, 34) and could be recently confirmed by computational fluid dynamics modeling (35). Our results underline the necessity to adjust nebulization characteristics post-operatively due to anatomical and functional ventilation changes depending on the extend of surgery.

Limitations of our study include using a cadaver head where the nasopharynx is firmly sealed by a silicon plug which is probably more efficient than closure achieved in a person who is asked to obstruct the pharynx with the soft palate. This might influence the measurements and lead to slightly better results than can be expected in real life.

Moreover, xenon gas is of higher viscosity than air. That could result in a systematic underestimation of sinus ventilation due to different ventilation time constants for xenon gas in comparison to normal air. On the other hand for therapeutic purposes, ventilation has to be achieved with an aerosolized drug with a droplet size that exceeds any gas molecule in size to be able to achieve a therapeutic effect. This might lead to a not quite as efficient ventilation of the sinuses as demonstrated with xenon. To overcome these problems, reduction of droplet size (1–3 μm) and improvements in the acoustic properties like changing frequencies, amplitudes, and nebulization flow rates have to be further investigated to increase the sinus drug deposition in the future (27, 36).

A further limitation of the study was that we were able to use only one specimen, what is not sufficient to generalize the results in a wide patient population with very heterogeneous sinus anatomy.

Finally, we were able to visualize and quantify the paranasal sinus ventilation by xenon-enhanced dynamic DECT using laminar and pulsating air-/gas flows in a cadaver model. The superiority of pulsating gas flow over laminar flows to achieve ventilation of the paranasal sinuses in the non-operated setting could be confirmed. FESS is highly effective in improving the ventilation of the sinuses and eliminating the need for pulsation in the postoperative setting. To evaluate the potential advantages of xenon-enhanced DECT for imaging sinus ventilation and to show that pulsating flow is also more efficient in drug delivery to the paranasal sinuses in comparison to the conventional nasal spray application, more patient studies in clinical settings are required.

## Data Availability Statement

The raw data supporting the conclusions of this article will be made available by the authors, without undue reservation.

## Ethics Statement

The studies involving human participants were reviewed and approved by Ethikkommission der Medizinischen Fakultät der Universität München, Pettenkoferstrasse 8a, 80336 München. Written informed consent for participation was not required for this study in accordance with the national legislation and the institutional requirements.

## Author Contributions

SB, WM, and US contributed to conception and design of the study. TJ organized and run the CT-scans. SB and MSa performed the statistical analysis. TH wrote the first draft of the manuscript. AB, MH, and MSc wrote sections of the manuscript. All authors contributed to manuscript revision, read, and approved the submitted version.

## Funding

The study was supported by Pari Pharma GmbH, Gräfelfing, Germany by providing the xenon supplying nebulization system.

## Conflict of Interest

The xenon supplying nebulization system used in the study was provided bei Pari Pharma GmbH, Gräfelfing, Germany. US is employee of Pari Pharma GmbH. The remaining authors declare that the research was conducted in the absence of any commercial or financial relationships that could be construed as a potential conflict of interest.

## Publisher's Note

All claims expressed in this article are solely those of the authors and do not necessarily represent those of their affiliated organizations, or those of the publisher, the editors and the reviewers. Any product that may be evaluated in this article, or claim that may be made by its manufacturer, is not guaranteed or endorsed by the publisher.
